# What do we know about the participation of hematopoietic stem cells in hematopoiesis?

**DOI:** 10.12688/f1000research.6459.1

**Published:** 2015-10-29

**Authors:** Nina Drize, Nataliya Petinati

**Affiliations:** 1 Federal Government Budget Institution National Research Center for Hematology, Ministry of Health, Moscow, Russian Federation

**Keywords:** Hematopoiesis, Stem cells, colony-forming units

## Abstract

The demonstrated presence in adult tissues of cells with sustained tissue regenerative potential has given rise to the concept of tissue stem cells. Assays to detect and measure such cells indicate that they have enormous proliferative potential and usually an ability to produce all or many of the mature cell types that define the specialized functionality of the tissue. In the hematopoietic system, one or only a few cells can restore lifelong hematopoiesis of the whole organism. To what extent is the maintenance of hematopoietic stem cells required during normal hematopoiesis? How does the constant maintenance of hematopoiesis occur and what is the behavior of the hematopoietic stem cells in the normal organism? How many of the hematopoietic stem cells are created during the development of the organism? How many hematopoietic stem cells are generating more mature progeny at any given moment? What happens to the population of hematopoietic stem cells in aging? This review will attempt to describe the results of recent research which contradict some of the ideas established over the past 30 years about how hematopoiesis is regulated.

## A piece of history

Nature and Nature’s laws lay hid in night:

God said, “Let Newton be!” and all was light.

Alexander Pope (1688–1744)

 

It did not last; the devil howling

“Ho! Let Einstein be!” restored the status quo.

Sir John Collings Squire (1884–1958)

Hematopoietic cells arise from mesodermal precursors in the developing vertebrate embryo in multiple waves in different anatomic sites: allantois, yolk sac, and the aorta-gonad-mesonephros ^1– 9^. Cells able to maintain lifelong hematopoiesis emerge first from ventral aortic hemogenic endothelial cells and immediately enter the circulation ^10– 13^. These hematopoietic stem cells (HSCs) home to and expand within the fetal liver, spleen, thymus and eventually seed the bone marrow—the major blood-forming organ in the adult ^3, 7^. The precise number of HSCs at all stages of development remains poorly defined.

The modern era of HSC characterization started with the work of Till and McCulloch ^14^. They described spleen colony-forming units (CFU-S). For many years thereafter, these cells were believed to have the properties of HSCs. Then it was discovered that most CFU-S can repopulate the hematopoietic system for only a short time and that they are descendants of phenotypically distinct cells that are able to repopulate the hematopoietic system for a long time ^15– 18^. The latter are usually detected by their ability to perpetually regenerate all blood cell types in a myeloablated recipient ^19^ and, in the adult, these HSCs are quiescent most of the time ^20^. Two general models have been put forth for how HSCs are recruited into proliferation and subsequent differentiation under normal physiological conditions. The “clonal succession model” proposes that small numbers of HSCs are sequentially recruited to leave the compartment and enter the cell cycle, and thereafter initiate an irreversible lineage commitment process ^16, 21– 23^. The “clonal stability model” suggests that randomly selected self-maintaining HSCs continuously replenish the supply of mature blood cells throughout an organism’s lifetime ^24– 26^.

## Methods of marking individual HSCs

In order to understand how normal hematopoiesis is maintained, we require methods that can trace the separate long-term outputs of individual cells. One approach has been to permanently and uniquely mark individual HSCs. Radiation-induced chromosomal markers were used by Till and co-authors to show that all cells in each spleen colony derive from a single cell ^27, 28^. However, the small number of chromosomally marked cells obtained using this approach made it inadequate to resolve the HSC clones operative in long-term transplant experiments.

The genetic marking of mouse HSCs *via* the retroviral-mediated transfer of a foreign gene provided evidence that one cell is capable of differentiating into all major types of hematopoietic cells ^29, 30^ and allowed the composition of lifelong clones derived from adult mouse bone marrow and fetal liver HSCs to be examined ^22, 31^. It was also shown that the hematopoiesis in the lethally irradiated mice transplanted with the marked cells was polyclonal and was supported initially by a large number of short-lived, successively active clones ^22, 32^. Moreover, it was found that the formation of blood is polyclonal, not only at the level of early pluripotent progenitor, CFUs, but also at the level of terminally differentiated mature cells ^33^.

Retroviral marking is, however, limited to cells that are actively cycling. This method thus fails to label any HSCs that are not dividing at the time of retroviral exposure. Early studies that used this method thus had low resolution, leading to an underestimation of HSC numbers ^34^. Moreover, the site where the retrovirus inserts into the host genome can have a major effect on the clonal output of the initially transduced HSC, ranging from a slight change to overt dominance and even leukemia ^35– 38^. These effects are reduced substantially if vectors are derived from lentiviruses, such as the human AIDS virus HIV ^39^, and the use of lentiviruses to track the progeny regenerated from initially quiescent mouse HSCs has revealed many more clones than found earlier using retroviral marking ^40^.

A breakthrough in the study of the repopulating potential of mouse HSCs occurred when the use of large libraries of lentiviruses encoding short, randomly varied DNA marker sequences (called barcodes) was introduced ^41, 42^. Upon integration, each such vector introduces a unique, identifiable, and heritable mark into the host cell genome, allowing the clonal progeny of each initially transduced cell to be tracked over time. By coupling the barcoding method to a high-throughput sequencing-based detection system, it became possible to identify even smaller clones ^42, 43^. The use of this sensitive methodology showed that many clones (about 70 per mouse) produced in mice transplanted with such marked cells can contribute to hematopoiesis over long periods of time, although their content of granulocytes, T cells, and B cells could be substantially different. Moreover, the contributions of individual clones to mature blood cells could change dynamically, with most clones either expanding or declining over time ^44^, and not necessarily in a fashion concordant with their activity in the bone marrow. Nevertheless, many clones were observed for more than 12 weeks. Thus, the regeneration of the hematopoietic system from transplanted cells can involve a contribution from a large number of input HSCs and progenitor cells, including self-renewing HSCs, as well as more differentiated and lineage committed progenitor cells.

The stromal microenvironment niches for HSCs also play an important role in hematopoiesis. It is known that the niches for the HSC differ in different places of hematopoiesis. It has been reported that there are fewer niches in the long bones than in trabecular bones, and the properties of the HSCs in the niches in these different sites are different ^45– 49^.

## Clonal composition of HSCs

Studies of the clonal composition of HSCs in primates have shown results that are generally similar to those obtained in mice, albeit with some substantial differences. Tracking of thousands of HSCs and progenitor cells in rhesus macaques for up to 12 years revealed that approximately half of the analyzed clones contributed to long-term repopulation (over 3–10 years), arising in sequential groups and likely representing self-renewing HSCs ^50^. Most of the remaining clones were observed only during the first year. A large number (43–71%) of clones were detected at an extremely low average frequency of <0.0002, contributing to <7% of total blood repopulation over the entire course of observation. The 5% of the most frequently detected clones contributed to an average of 49–72% of all regenerated blood cells, depending on the animal and cell type. About 330 clones per animal were detected. The sequential expansion of different groups of clones (over several months for the earliest clones and over several years for those clones appearing later after repopulation) was revealed. Approximately equal numbers of long-term and short-term clones were detected. Although the assay endpoint given to each animal to distinguish long-term and short-term clones ranged from 3–10 years, this variation in endpoints did not significantly affect the findings regarding the relative frequencies of these two populations in so far as the clonal kinetics in all animals became more stable after 1–2 years ^50^.

In humans, HSC kinetic data derived from the clonal tracking of their activity *in vivo* have been obtained from gene therapy clinical trials for adenosine deaminase (ADA) deficient-SCID and Wiskott-Aldrich syndrome (WAS). These trials involved the infusion of genetically engineered HSCs whose progeny could then be followed over time by tracking the unique barcode integration sites (ISs) of the therapeutic vectors incorporated into the transplanted cells. Authors analyzed the timing of short, intermediate, and long-term HSC output showing that CD34+ clones active at 3–6 months after transplantation were not detectable at later times ^51^. In the later steady-state, about 200 clones per person was the figure estimated by mark-recapture of transduced HSC clones that were stably contributing to the progenitor’s repertoire for up to 3 years after infusion of gene-corrected CD34+ cells. To evaluate the long-term preservation of activity by the transplanted HSCs, the authors exploited data derived from the IS-based tracking of 4,845 clones in ADA-SCID patients performed for up to 6 years after gene therapy. This analysis showed that identical ISs were consistently detected in multiple lineages at stable levels even several years after transplantation. Strikingly, semi-quantitative PCR used to measure specific vector-genome junctions revealed a fluctuating, but consistent, output of marked clones over a period of 5 years without evidence of transient inactivity. Additionally, since the gamma-retroviral vector used in ADA-SCID HSC-GT trial is able to transduce only actively replicating cells, these results provided the first evidence that *in vitro* activated human HSCs can still display long-term activity *in vivo* ^51, 52^. Thus, in humans and primates, hundreds of individual clones could be shown to contribute to different cell lineages with clonal stability established after an initial phase of instability during the first year after transplantation.

In all previously described transplantation studies, the progeny of individual HSCs were tracked using an *ex vivo* viral transduction of the cells prior to transplantation into myelosuppressed hosts. This approach is not effective for labeling HSCs *in situ*. To reduce the drawback of studying the behavior of cells stimulated to repopulate myeloablated recipients, mice were sublethally irradiated without hematopoietic cell transplantation. Day 10 CFU-S assays of bone marrow cells subsequently sampled throughout a 12– to 20–month period revealed chromosomally marked clones that remained active for at least 1.5 years of the life of the mice ^53, 54^. These long-lived clones produced 10% of all identified CFU-S. Interestingly, studies of immunophenotypically defined HSCs showed that only 10% of those that divided were able to return to a resting state ^55^. In summary, some HSCs are able to maintain hematopoiesis for long periods and can return to a quiescent state after transient proliferative activity.

Recent approaches used to track cells using a transposon system cloned into mice ^56^ have now provided an opportunity to study the control of endogenous hematopoiesis without the use of transplantation ^57^. The transposon is activated by a hyperactive “sleeping beauty” transposase whose expression is controlled by doxycycline. During the short time period when doxycycline is applied to a mouse, the transposon can randomly mobilize to a different genomic location. This transposition creates an inheritable genomic DNA insertion that is unique to individual cells and their progeny. Cells originating from a common ancestor can thus be identified by their shared unique transposon IS. The transposon system has produced strikingly different results than those from previous studies. At each of their measured time points, dramatically different clones appear to supply the blood. The authors estimated that thousands of clones contribute to blood formation at any time point. Thus, their data suggest that long-term hematopoiesis is sustained by the successive recruitment of a large number of clones. While their observed clonal dynamics are consistent with the “clonal succession model”, their estimated clonal complexity is much greater than could possibly be supported by the relatively small number of HSCs traditionally identified using transplantation methods. The clonal composition of hematopoietic cells derived from clonally marked donor mice when compared with that of transplanted recipients revealed that donors and recipients possessed different clonal repertoires. The authors also compared the clonal composition of the HSC compartment with that of the intermediate progenitors, and the mature blood cells present in individual mice. They found that fewer than 5% of HSC clones are subsequently represented in mature cell populations, whereas the clonal composition of the multipotent progenitor and myeloid progenitor populations did mirror the mature cell populations. Based on these two experiments, the authors conclude that the cells that supply blood under homeostatic conditions are not transplantable and are not found in the conventionally defined HSC pool. Moreover, they suggest that the large number of progenitor cells, including previously defined multipotent progenitors and myeloid progenitors, may be the major source of ongoing hematopoiesis. These data imply a need to change our idea of the regulation of steady-state hematopoiesis. However, it should be noted that these data do not exclude the possibility that HSCs participate in steady-state blood production but, if they do, they must be quickly depleted from the HSCs pool once they committed to differentiate.

## What happens during aging

The dynamics of hematopoiesis depend on the phase of ontogenesis from the cradle to the grave. What happens to the pool of HSCs during aging? The number of transplantable HSCs increases in the bone marrow of old mice ^58– 60^, although clonal analysis of their progeny has revealed many functional defects ^61^. For example, the clonal outputs of HSCs from young mice are, on average, larger than those produced by HSCs from old mice ^44^. These data appear to contradict the recent results of whole-exome sequencing of DNA in peripheral blood cells from aging humans ^62, 63^, where the development of oligoclonal hematopoiesis was found to be a relatively common condition and one associated with an increased risk of hematologic cancer. Based on deep whole-genome sequencing, it was estimated that approximately 450 somatic mutations had accumulated in the nonrepetitive portions of the genome present in healthy blood cells in a 115-year-old woman. The distribution of variant allele frequencies of these mutations suggests that the majority of the peripheral white blood cells were the offspring of two related HSC clones. Moreover, the telomere lengths of the white blood cells were significantly shorter than the telomere lengths from other tissues. Together, this suggests that the finite lifespan of HSCs, rather than somatic mutation effects, may lead to hematopoietic clonal evolution at extreme ages ^64^. Exhaustion of the HSC pool leading to a reduced number of functioning clones might explain how such an oligoclonal picture of hematopoiesis develops in humans. It is thus becoming increasingly clear that what we know about the structure and functioning of the hematopoietic system is still just the tip of the iceberg, with much more to understand that is still under water.
