# Supplementary material for: What do we know about the participation of hematopoietic stem cells in hematopoiesis?
Source: F1000Res. 2015 Oct 29;4:F1000 Faculty Rev-1177. [Version 1] doi: 10.12688/f1000research.6459.1 (PMC4815613; doi:10.12688/f1000research.6459.1)
Supplement: Peer review history

# Peer review: Referee response for version 1

**DOI:** 10.5256/f1000research.6931.r10984

**Published:** 2015-10-29

**Authors:** Leonid Bystrykh

**Affiliations:** 1 European Institute for the Biology of Ageing (ERIBA), Section Ageing Biology and Stem Cells, University of Groningen, Groningen, Netherlands

This review is an overview of the hematopoietic stem cells in hematopoiesis, and it is my opinion that there are still some issues with the review. For instance, I think that the abstract is not fully aligned with the body of the review and instead I feel that it should summarise the major statements made in the text. Moreover, in the main body of the review, there are a number of factual inaccuracies and the authors present the evolution of our knowledge as a series of mistakes which, in my view, is incorrect and they have not provided sufficient information on the methods and tools that they describe. I miss a more critical expert review of the evolution of our tools to study hematopoietic stem cells. As a result of these issues, I have given this review an “Approved with Reservations” rating.

I have read this submission. I believe that I have an appropriate level of expertise to confirm that it is of an acceptable scientific standard, however I have significant reservations, as outlined above.

# Peer review: Referee response for version 1

**DOI:** 10.5256/f1000research.6931.r10983

**Published:** 2015-10-29

**Authors:** Hector Mayani

**Affiliations:** 1 Oncology Research Unit, Oncology Hospital, National Medical Center, IMSS, Mexico City, Mexico

I have read this submission. I believe that I have an appropriate level of expertise to confirm that it is of an acceptable scientific standard.

# Peer review: Referee response for version 1

**DOI:** 10.5256/f1000research.6931.r10982

**Published:** 2015-10-29

**Authors:** Connie Eaves

**Affiliations:** 1 Terry Fox Laboratory, British Columbia Cancer Agency, Vancouver, BC, Canada

I have read this submission. I believe that I have an appropriate level of expertise to confirm that it is of an acceptable scientific standard.
